# High Glucose Concentrations Impair the Processing and Presentation of *Mycobacterium tuberculosis* Antigens In Vitro

**DOI:** 10.3390/biom11121763

**Published:** 2021-11-25

**Authors:** Guadalupe Monroy-Mérida, Silvia Guzmán-Beltrán, Fernando Hernández, Teresa Santos-Mendoza, Karen Bobadilla

**Affiliations:** 1 Laboratory of Immunopharmacology, Instituto Nacional de Enfermedades Respiratorias “Ismael Cosío Villegas”, Mexico City 14080, Mexico; gmonroymerida@yahoo.es (G.M.-M.); tesalonster@gmail.com (T.S.-M.); 2 Department of Microbiology, Instituto Nacional de Enfermedades Respiratorias “Ismael Cosío Villegas”, Mexico City 14080, Mexico; guzman.silvia@gmail.com; 3 Research Department of Virology and Micology, Instituto Nacional de Enfermedades Respiratorias “Ismael Cosío Villegas”, Mexico City 14080, Mexico; fhernndezsnchez@gmail.com

**Keywords:** high glucose concentrations, mycobacterium tuberculosis, antigenic processing, antigenic presentation, CD4+ T cells

## Abstract

Type 2 diabetes is an established risk factor for tuberculosis, but the underlying mechanisms are largely unknown. We established an in vitro model to analyze the effect of high glucose concentrations in antigen processing and presentation in antigen-presenting cells. Human monocyte-derived macrophages (MDMs) were exposed to high (11 mM and 30 mM) and low (5.5 mM) glucose concentrations and infected with *Mycobacterium tuberculosis (Mtb)*. Flow cytometry was used to analyze the effect of high glucose concentrations in histocompatibility complex (MHC) class II molecules (HLA-DR) and co-stimulatory molecules (CD80 and CD86), indispensable for an adequate antigenic presentation and CD4+ T cell activation. HLA-DR and CD86 were significantly decreased by high glucose concentrations compared with low glucose concentrations. Confocal microscopy was used to detect Rab 5 and Lamp-1, proteins involved in the kinetics of antigen processing as early markers, and Rab 7 and cathepsin D as late markers. We observed a delay in the dynamics of the acquisition of Rab 7 and cathepsin D in high glucose concentrations. Moreover, the kinetics of the formation *M. tuberculosis* peptide–MHC II complexes in MDMs was decreased under high glucose concentrations, reducing their capacity for T cell activation. These findings suggest that high glucose concentrations directly affect antigenic processing, and therefore antigenic presentation.

## 1. Introduction

Type 2 diabetes (T2D) is characterized by abnormally elevated levels of blood glucose due to impaired insulin secretion and glucose intolerance [1,2]. T2D is associated with an increased risk of several infections, including tuberculosis (TB), which is the most significant cause of death from an intracellular bacterial infection worldwide [3]. It is estimated that up to one-third of the global population is currently infected with *Mycobacterium tuberculosis,* the TB causal agent [4].

The combination of T2D and TB is still a global challenge, not only because the prevalence of T2D is higher in populations that also show increased TB infection rates, but also because diabetic patients tend to develop a more severe TB disease [5,6].

Although CD4+ T cell responses are known to be relevant in the control of TB, the immune mechanisms underlying TB susceptibility in diabetic patients remain unclear. Antigen internalization, processing, and presentation by antigen-presenting cells (APCs) play a role in triggering the CD4+ T cell-dependent responses, which include the production of IFN-γ to optimize macrophage intracellular killing [7,8]. In this sense, phagosome maturation is critical for proper antigenic processing and the formation of peptide–MHC class II complexes [9,10].

Antigen processing involves several sequential steps, starting with (1) the internalization of pathogens and their products via phagocytosis, followed by (2) the catabolism of proteins into peptide fragments through a process known as phagosome maturation, characterized by continuous and dynamic fusion and fission events with transport vesicles, early and late endosomes, and lysosomes [9,11,12]. Several proteins, including the Rab GTPases, Rab 5 and Rab 7, and enzymes such as cathepsin D, acidic hydrolases, and lysosome-associated membrane proteins (Lamp-1) participate in phagolysosome biogenesis. Rab 5 and Lamp-1 are associated with phagosomes immediately after phagocytosis, and Rab 7 appears on the phagosome membrane after Rab 5 dissociation and resides on the membrane during phagosome maturation. Finally, the acquisition of cathepsin D favors phagolysosome maturation [13,14,15,16]. The last step is the binding of mycobacterial peptides to intracellular MHC II molecules and the trafficking of these complexes to the plasma membrane for presentation to CD4+ T cells. As mentioned above, high blood glucose levels are the hallmark of T2D, and there is evidence that excess glucose can directly impair immune functions such as T cell activation, cytokine production, and antigen processing [17,18,19].

In this sense, high glucose concentrations inhibit lectin binding, thereby contributing to poor pathogen recognition and impaired phagocytosis in macrophages and monocytes from diabetic patients with poor glycemic control [20,21,22]. At the same time, *M. tuberculosis* limits the acquisition of Rab 7, delaying phagosomal maturation and favoring the persistence of Rab 7 within the macrophages [23,24].

In addition, some reports indicate an initial delay in the activation of Th1 cell-mediated immunity in experimentally induced hyperglycemia models in mice, and a lower production of IFN-γ by CD4+ T cells from patients with TB and high blood glucose [18,25,26].

The foregoing lines of evidence suggest an impairment of the innate and adaptive immunity in T2D and TB, wherein high glucose levels may directly alter the processing and presentation of antigens and T cell activation through mechanisms that remain unknown.

The main goal of this study was to investigate the effect of high glucose concentrations on the ability of APCs to process *M. tuberculosis* and stimulate IFN-γ release in CD4+ T cells. For this purpose, we used an in vitro model with three different glucose concentrations: 5.5 mM (~99 mg/dL) to simulate conditions in nondiabetic individuals, 11 mM glucose (~198 mg/dL) for T2D patients with acceptable glycemic control, and 30 mM glucose (~540 mg/dL) for patients with hyperglycemic crises due to uncontrolled diabetes [27,28].

## 2. Materials and Methods

### 2.1. Bacterial Culture

*Mycobacterium tuberculosis* H37Ra was purchased from the American Type Culture Collection (ATCC 25177, Rockville, MD, USA). It was grown at 37 °C in 7H9 broth (Difco, Detroit, MI, USA) supplemented with ADC (Beckton Dickinson [BD], San Jose, CA, USA) and 1% glycerol (Sigma-Aldrich, MO, USA). The bacterial concentration was determined by counting colony-forming units on Middlebrook 7H10 agar plates (BD) and supplemented with OADC (BD) and 1% glycerol. Before infection of the human cells, *M. tuberculosis* was disaggregated to achieve a single-cell suspension. For this purpose, a bacterial stock was thawed and centrifuged at 3000× *g* for 8 min and washed in RPMI 1640 medium (Lonza, Basel, Switzerland) without antibiotics. The bacterial suspension was subsequently passed through a 26-gauge needle, followed by sonication at 80 cycles for 30 s. Residual clumps were removed via centrifugation at 500× *g* for 1 min. To improve phagocytosis, bacteria were incubated for 30 min in RMPI medium supplemented with 10% pooled human serum without antibiotic at 37 °C before incubation with cells.

### 2.2. High-Glucose Model In Vitro and Human Monocyte-Derived Macrophages (MDMs) and Dendritic Cells (DCs)

MDMs and dendritic cells (DCs) were obtained from peripheral blood mononuclear cells (PBMCs) through the centrifugation of buffy coats from healthy blood bank donors at the Instituto Nacional de Enfermedades Respiratorias under approbation of the Institutional Ethical Review Board (Protocol B14-19).

Heparinized blood was diluted 1:2 with RPMI 1640 layered on lymphocyte separation solution (Lonza) and centrifuged at 300× *g* for 45 min at room temperature. The PBMCs were harvested, and monocytes were positively selected using CD14^+^ immunomagnetic beads (Miltenyi Biotec, Auburn, CA, USA) according to the manufacturer’s instructions. For MDM differentiation, monocytes were cultured with GM-CFS (10 ng/mL) on days 1, 3, and 5. For DCs differentiation, monocytes were cultured with GM-CSF (50 ng/mL) and IL-4 (25 ng/mL) on days 1 and 4 until day 5, when immature DCs (iDC) were obtained (At this point, the cells had acquired macrophage or dendritic cell morphology and called MDMs or DCs, respectively.

#### High Glucose Stimulation

Monocytes (to be differentiated to MDMs and DCs) were incubated for 7 days in glucose-free RPMI-1640 medium containing 10% FBS, 2 mM l-glutamine, and 5.5 mM (99 mg/dL) of D-glucose, designed to resemble normal glucose levels observed in healthy subjects. High glucose monocytes were incubated in glucose-free RPMI-1640 medium containing 10% FBS, 2 mM l -glutamine, and 11 mM or 30 mM (198 mg/dL and 540 mg/dL, respectively) for 7 days to resemble glucose levels in diabetic patients with controlled or uncontrolled hyperglycemia, respectively. Control osmotic pressure was achieved by incubating MDM in glucose-free RPMI-1640 medium containing 10% FBS, 2 mM l-glutamine, and D-mannitol at 37 °C in a 5% CO_2_ atmosphere for 7 days.

### 2.3. WST-1 Viability Assay

The metabolic activity was monitored by the colorimetric WST-1 assay of the mitochondrial dehydrogenases according to the manufacturer’s instructions (11644807001; Roche Applied Science, Rotkreuz, Switzerland). MDMs were cultured with different concentrations of glucose (described above) and mannitol at 37 °C in a 5% CO_2_ for 7 days. The WST-1 cleavage product was measured at day 1, 3, 5, (data no shown), and 7 of culture at 650 nm in a multimode microplate reader (Synergy HT Biotek, Vermont, USA). WST-1 plus medium alone served as a blank, which was subtracted from all values. Percentages of WST-1 activity were calculated by the following formula: (WST-1 value/diluent control) 100.

### 2.4. Antibodies

Confocal microscopy: Rabbit anti-Rab 5 (C8B1), anti-rabbit F (ab′)2 Alexa-Fluor 647, rabbit anti-Rab 7 (D95F2) XP, anti-rabbit (ab′)2 Alexa-Fluor 647, and mouse anti-Lamp-1 (D401S) were purchased from Cell Signaling Technology (Danvers, MA, USA). Donkey anti-mouse Cy^TM^3 Affinity pure and donkey anti-goat Alexa Fluor 594 Affinity pure were purchased from Jackson Immune Research (West Grove, PA, USA). Rabbit anti-cathepsin D (E179) was purchased from Cell Signaling Technology (Danvers, MA, USA).

Cell cytometry assays: FITC HLA-DR (clone G46-2.6; BD Pharmingen FL, New Jersey, USA), APC mouse anti-human CD86 clone 2331, and FITC anti-human CD80 clone L307.4 were purchased from Biosciences Pharmingen (San Jose, CA, USA).

### 2.5. CD4+ T-Lymphocyte Proliferation Assays

PBMCs were resuspended in serum-free RPMI medium with 0.5 mL of 5 mM 5(y6)-carboxyfluorecein diacetate succimidyl ester (CFSE) (Affymetrix Thermo Fisher Scientific, Waltham, MA, USA) and incubated for 10 min at 37 °C in agitation. Unconjugated dye was removed with fetal bovine serum. Immediately, CFSE-labeled CD4+ T cells were resuspended in 5.5 mM and 30 mM glucose and stimulated with anti-CD3/CD28 beads (Miltenyi Biotec, Auburn, CA, USA) (4:1) for 6 h. The beads were then removed, and the cells were maintained in culture with IL-2 (25 U/mL) for 3 days. Cells were then harvested and labeled with mouse anti-human CD4+ APC-Cy7 (BD Biosciences, San Jose, CA, USA). The percent of T cell proliferation was determined by CFSE dilution compared with initial fluorescence at day of staining, using flow cytometry (BD Facs Aria II cytometer, BD Biosciences), and analyzed by FlowJo software.

### 2.6. Detection of HLA-DR, CD80, and CD86 Molecules by Flow Cytometry

MDMs were cultured with 10 ng/mL of GM-CSF in culture medium with 5.5 mM and 30 mM glucose at 37 °C in a 5% CO_2_ atmosphere for 7 days. For intracellular staining, 5 X 10^5^ cells per tube were washed by centrifugation (washing buffer: 0.1% BSA in PBS) and supernatant was discarded. Cells were permeabilized with 150 μL of permeabilization/fixation buffer Invitrogen (Carlsbad, CA, USA) and incubated at 4 °C for 30 min. Then, cells were washed and incubated in 100 μL of Abs solution (1:100) at room temperature for 30 min, washed twice, and resuspended in 200 μl of 1% paraformaldehyde. Samples were stored at 4 °C protected from light until acquisition. FITC mouse anti-human HLA-DR (clone G46-2.6; BD Pharmingen, Franklin Lakes, NJ, USA).

For surface staining, 24 h after stimulation with lipopolysaccharide (100 ng/mL), the cells were harvested (5 × 10^5^ cells per tube) and washed. Blocking solution (200 μL of 1% BSA in PBS) was added and the cells were incubated in 100 μL of Abs solution (1:100) Anti-human Abs APC CD86, FITC CD80 at 4 °C for 20 min, and then were washed and resuspended in 200 μL of 1% paraformaldehyde. Samples were stored at 4 °C protected from light until acquisition. In addition, in another tube without stimulus, cells were incubated in 100 μL of Abs solution (1:100) FITC HLA-DR 4 °C for 20 min. Data were acquired (at least 10,000 events per sample in MDMs gate) on FACS ARIA II (Becton Dickinson, Franklin Lakes, NJ, USA) flow cytometer and analyzed using Flow Jo software.

### 2.7. Detection of Proteins of the Phagolysosomal Pathway by Confocal Microscopy

For the microscopy experiments, 1 × 10^5^ cells/well in 500 μl of RPMI medium were cultivated in an 8-well Lab-Tek II chamber and incubated in both 5 mM and 30 mM glucose concentrations for 7 days at 37 °C and 5% CO_2_. Then, phagocytosis of *M. tuberculosis* previously stained with PKH67 Green Fluorescent Cell Linker Midi Kit (Sigma-Aldrich, St. Louis, MO, USA) was induced for 1 h at 37 °C and 5% CO_2_. Phagocytosis was stopped on ice and the cells were washed with PBS three times to remove noninternalized bacteria and incubated at 37 °C, 5% CO_2_ in different times of early antigenic processing, 30, 60, 120 min, and late antigenic processing, 60, 120, and 480 min, respectively. Finally, MDMs were fixed with paraformaldehyde 1%, washed, and blocked.

After incubation with primary and secondary antibodies, cells were washed and the coverslips were mounted with ProLong Gold antifade mounting (Invitrogen, Life Technologies, Carlsbad, CA, USA) on glass slides. Images were viewed with an Olympus Fluoview FV-1000 laser scanning confocal microscope. Images were quantified using the ImageJ software (NIH, Bethesda, MD, USA).

### 2.8. Amplification of M. tuberculosis-Specific CD4+ T Cells

Autologous CD4+ T-specific cells for *M. tuberculosis* were amplified from the PBMCs of tuberculin skin test positive donors, as described by Bobadilla et al. [29]. Briefly, PBMCs (1 × 10^6^ cells/well) were cultured in 24-well plates and stimulated with live *M. tuberculosis* at a multiplicity of infection of 1:10. On days 3, 5, and 7, cultures were supplemented with 50 U/mL of IL-2 [30]. After 7 days, expanded CD4+ T cells specific for mycobacterial antigens were purified by positive selection using CD4+ human microbeads (MACS Miltenyi Biotec, Auburn, CA, USA) according to the manufacturer’s instructions. The mean purity of positively selected CD4+ cells was 96 ± 2%, as determined by flow cytometry. For each experiment, the specific autologous CD4+ T cells were cocultured with autologous MDMs infected with *M. tuberculosis*, tetanus toxoid (data no shown), or uninfected cells. Interferon-γ production was detected by ELISA.

### 2.9. Detection of M. tuberculosis Peptide–MHC II Complexes and Determination of M. tuberculosis-Processing Kinetics in Different Concentrations of Glucose

MDMs were cultured in 96-well flat bottom plates (1.5 × 10^5^ cells/well) in one low glucose concentration (5.5 mM) and two high glucose concentrations (11 mM and 30 mM). After 7 days in culture, the MDMs were infected with *M. tuberculosis* at a multiplicity of infection of 10. To ensure maximal phagocytosis, the cells were incubated for 1 h and washed with ice cold RPMI 1640 to remove any extracellular bacteria. Prewarmed medium was added, and the cells were incubated at 37 °C for different periods of time (chase time) ranging 30 to 120 min. Antigen processing was stopped by fixing the MDMs with 1% paraformaldehyde, as previously described [29,31]. The expression of the peptide–MHC II complexes on the plasma membrane of the fixed MDMs was detected by incubation with bacterial-specific CD4+ T cells (1 × 10^5^ cells/well), and IFN-γ release was measured after 24 h. ELISA was used to detect IFN-γ in the culture supernatants using an IFN-γ kit according to the manufacturer´s instruction Biolegend (San Diego, CA, USA). The absorbance was measured in a Synergy HT Biotek multimode microplate reader at 405 nm.

## 3. Results

### 3.1. High Glucose Concentrations Did Not Affect the Cell Viability in an In Vitro Model

**Figure 1:**
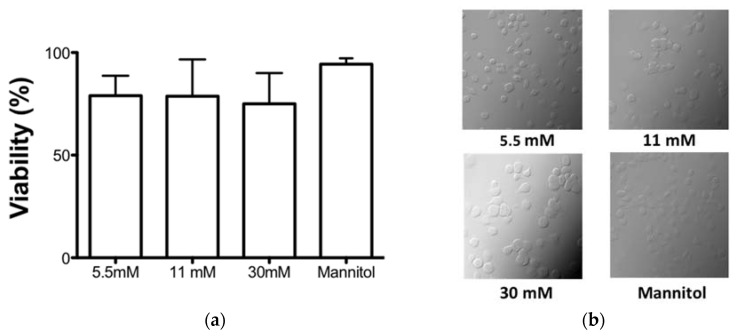
Effect of glucose concentrations on cell viability. The cells were treated in the presence of 5.5, 11, and 30 mM glucose and mannitol for 7 days. (**a**) The viability was quantified by WST-1 reduction and the number of viable cells is expressed as the percent of reduction of WST-1 compared with the control. Data were expressed as mean SEM from four independent experiments. (**b**) Representative phase contrast micrographs showing the effect of different glucose concentration or mannitol in human cells.

We established an in vitro model to evaluate the effect of high glucose concentrations on the functions of the APCs. We evaluated the effect of 5.5 mM, 11 mM, and 30 mM of glucose and mannitol as an osmolarity control on the cell viability of the MDMs after 7 days of culture. We observed no differences in the viability of the MDMs in any of the treatments (Figure 1a). In addition, we verified the morphology in bright field micrographs, and we observed that the MDMs were round and bright throughout the field with all of the treatments after 7 days of culture (Figure 1b).

### 3.2. CD4+ T Cell Proliferation Was Maintained despite the High Glucose Concentrations

**Figure 2:**
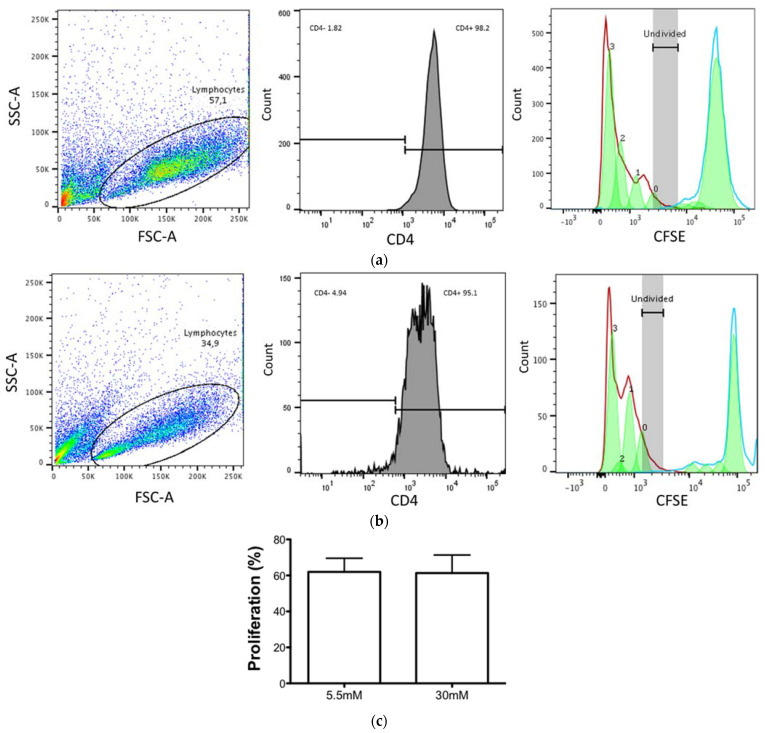
Proliferative capacity of CD4+ T cells is unaffected by high glucose concentrations. PBMCs were incubated in 5.5 mM and 30 mM glucose and stimulated with anti-CD3/CD28 beads for 6 h and maintained in culture with IL-2 (25 U/mL) for 3 days when the cells were stained for CD4 detection. (A) Representative dot plot showing the gating strategy for lymphocyte population (left). Histogram showing CD4+ T cells population (APC Cy7) from lymphocyte gate (middle panel). Histogram of CFSE proliferative assay of CD4+ T cells unstimulated (blue line) and stimulated with anti-CD3/CD28 (red line), 3 generations (right panel), in 5.5 mM (**a**) and 30 mM (**b**). (**c**) The percentage of CD4+T cell proliferation was unaffected by high glucose concentrations. Data were analyzed using a *paired t test*, and no significant differences were observed. Data are expressed as mean ± standard deviation from three independent experiments.

The recognition of the *M. tuberculosis*–MHC II peptide complexes on the membrane of APC by CD4+ T cells is the first step for the generation of an effective and specific immune response. Therefore, we next evaluated the effect of high glucose concentrations on the CD4+ T cell proliferation capacity induced by TCR stimuli (antiCD3/antiCD28) and on the unstimulated lymphocytes. The PBMCs were stained with CFSE, stimulated, and incubated for three days to measure the proliferation by CFSE dilution. As shown in Figure 2, we observed no differences in T lymphocyte proliferation between 5.5 mM glucose (a, c) and 30 mM glucose (b, c). These results indicate that the intrinsic capacity of CD4+ T cells to proliferate is unaffected by high glucose concentrations.

### 3.3. High Glucose Concentrations Decreased MHC Class II and CD86 Molecules in MDMs

**Figure 3:**
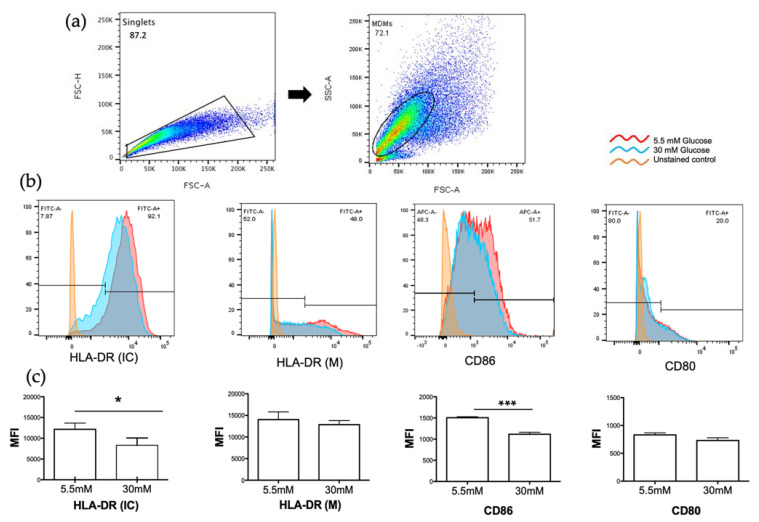

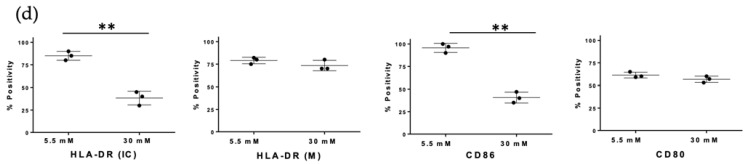
Detection of MHC class II (HLA-DR) and co-stimulatory molecules (CD86, CD80) in 5.5 and 30mM of glucose. Monocyte derived macrophages (MDMs) were gated for singlets on a forward scatter height/forward scatter area density plot (**a**, left). MDMs were then gated on a side scatter area/forward scatter area density plot (**a,** right) for detection of intracellular HLA-DR (HLA-DR IC), membrane HLA-DR (HLA-DR M), CD86, and CD80. Histograms of each marker of a representative experiment are shown (**b**). (**c**) MFI of the indicated markers were measured; mean ± SD three independent experiments are shown. (**d**) Percentage of cells expressing each marker. Data were analyzed using unpaired t test. Differences were considered significant when *p* < 0.05 with asterisks as follows: * *p* < 0.01, ** *p* < 0.003 and *** *p* < 0.0003. MFI, mean fluorescence intensity.

MHC class II and co-stimulatory molecules (CD80, CD86) play a critical role in the induction of immune response by presenting peptides of foreign antigens to CD4+ T lymphocytes, which results in their activation and proliferation. We analyzed the effect of 5.5 mM glucose or 30 mM glucose on the surface expression of MHC II, CD80, and CD86 molecules on the MDMs, as well as intracellular MHC II molecule expression by flow cytometry. As shown in Figure 3, CD80 and MHC class II (HLA-DR) molecules on the surface of MDMs were similar in both conditions (b, c). However, the mean fluorescent intensity of intracellular MHC class II (HLA-DR) molecules was decreased in 30 mM glucose with a statistically significant difference (*p*= < 0.01) when compared with 5.5 mM glucose (Figure 3b,c). Notably, the mean fluorescent intensity of CD86 was also reduced in high glucose concentrations (*p*= < 0.0003) versus low glucose concentrations (Figure 3b,c).

### 3.4. Rab 5 and Lamp-1 Are Retained in High Glucose Concentrations

**Figure 4:**
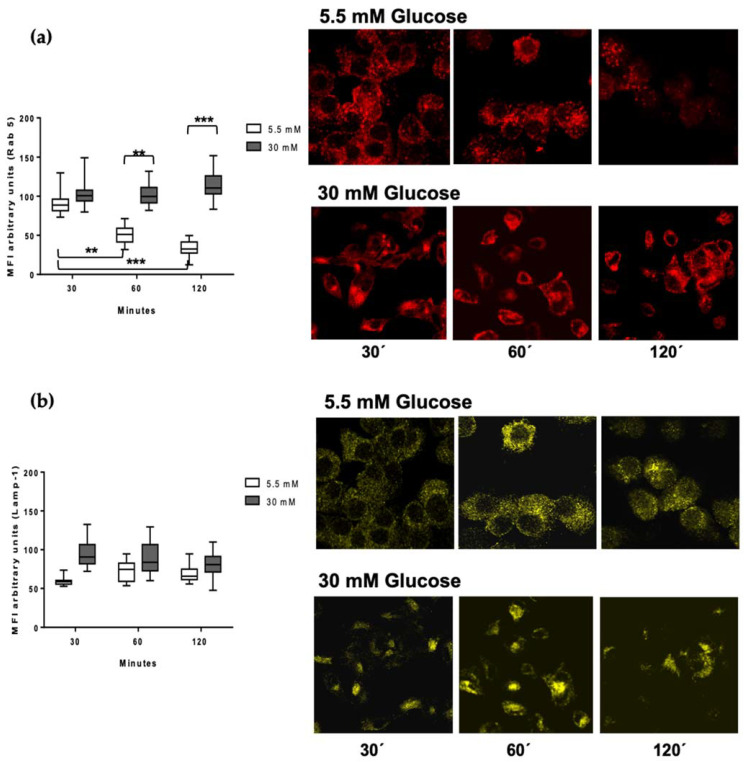
Detection of Lamp-1 and Rab 5 in the processing kinetics of *M. tuberculosis* in 5.5 and 30 mM of glucose. MDMs were infected with *M. tuberculosis* previously stained with PKH67 green fluorescent for one hour and incubated at 37 °C, 5% CO_2_, during 30, 60, and 120 min. (**a**) Rab 5 (red) and (**b**) Lamp-1 (yellow) quantification of mean fluorescence intensity from at least 100 cells from each condition (5.5 mM and 30mM of glucose) and time point using the IMAGE J software. A representative confocal microscopy image of each condition is shown. Data were analyzed using two-way ANOVA, and the differences were considered significant when ** *p* < 0.001, *** *p* < 0.0001; from four independent experiments. Confocal image 60×, 1 zoom.

Antigen processing involves the internalization of pathogens and their products via phagocytosis, the catabolism of proteins to peptide fragments, the binding of peptides to intracellular MHC class II molecules, and the trafficking of these complexes to the plasma membrane for presentation to CD4+ T cells. In order to analyze the regulation of early endosomal proteolysis of *Mtb* in the MDMs, we selected the Rab 5 protein as a marker of early phagosomes, and the lysosome-associated membrane protein LAMP-1 as a marker of a late phagosomal stage. After bacterial infection of the MDMs, we performed kinetics at different processing times of 30, 60, and 120 min, and detected Rab 5 (red) and Lamp-1 (yellow) by confocal microscopy (Figure 4a,b). In 5.5 mM glucose, we observed the presence of Rab 5 at 30 min of processing, but it was lost over time, in concordance to its reported dynamics in intervesicular traffic (Figure 4a) [14]. Conversely, at 30 mM glucose, Rab 5 remained constant along the kinetic timeline, indicating delayed dynamics (Figure 4a). To confirm this effect of high glucose concentrations, we quantified the MFI of the Rab 5 signal. The graphic shows the significant difference between 5.5 vs. 30 mM glucose at 60 (*p* < 0.001) and 120 min (*p* < 0.0001) processing times (Figure 4a). The presence of Lamp-1 is evident starting at 30 min and is maintained until 120 min (Figure 4b). In high glucose concentrations, we observed the maintenance of Lamp-1 from 30 min to 120 min in low glucose concentrations, as is shown in the graphic in which no statistical differences are observed (Figure 4b) [32].

### 3.5. Delayed Acquisition of Rab 7 and Cathepsin D in High Glucose Concentrations

**Figure 5:**
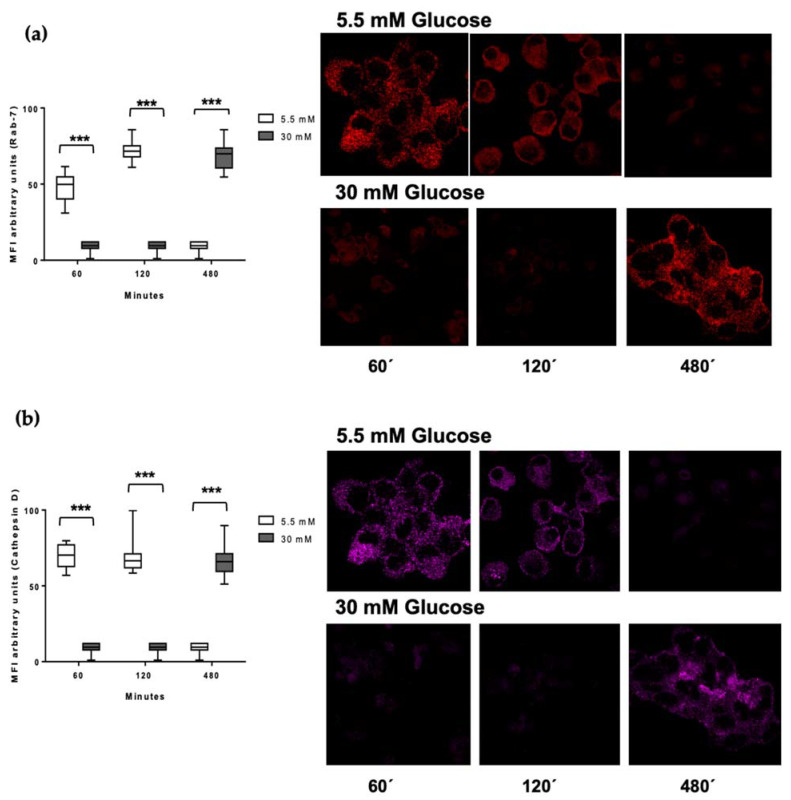

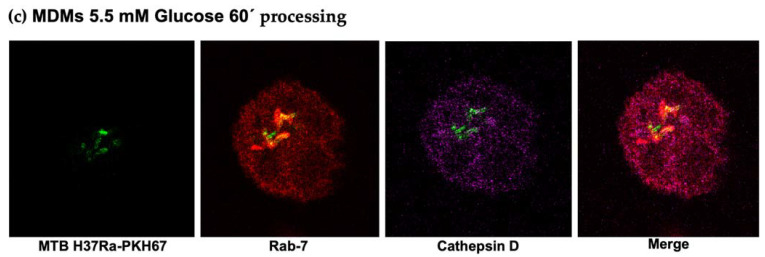
Detection of Rab7 and cathepsin D in the processing kinetics of *M. tuberculosis* in 5.5 and 30 mM of glucose. MDMs were infected with *M. tuberculosis* previously stained for one hour with PKH67 green fluorescent and incubated at 37 °C, 5% CO_2_ in a time kinetics (60, 120, and 480 min). (**a**) Rab 7 (red) and (**b**) cathepsin D (magenta) quantification of mean fluorescence intensity from at least 100 cells from each condition (5.5 mM and 30mM of glucose) in the kinetic sequence. A confocal microscopy image representative of each condition is shown. Data were analyzed using two-way ANOVA and the differences were considered significant when *** *p* < 0.0001; from four independent experiments. Confocal image 60×, 1 zoom. (**c**) Representative image of a reconstruction of Z sections (15 micrometer) and stack of each section of Mtb PKH67 green fluorescent (green), Rab 7 (red), cathepsin D (magenta).

We decided to perform the kinetics of *M. tuberculosis* processing in late phases of phagolysosomal maturation. We monitored the Rab 7 protein, which is important in directing phagolysosomal trafficking, and cathepsin D, one of the lysosomal enzymes whose function is essential to obtain mycobacterial peptides needed to form the *Mtb*–MHC II complexes. Figure 5 shows the acquisition of Rab 7 (a) and cathepsin D (b) in 5.5 and 30 mM glucose. Both molecules are present from 60 min to 120 min but show a decrease at longer times (480 min of processing) in 5.5 mM glucose. Notably, in high glucose concentrations, the acquisition of Rab 7 and cathepsin D was undetected at 60 and 120 min, and both molecules were detected until 480 min of processing (Figure 5). To confirm this effect of high glucose concentrations, we quantified the MFI of the Rab 7 and cathepsin D signal. The graphic shows the significant difference between 5.5 vs 30 mM glucose at 60, 120, and 480 min (*p* < 0.0001) processing times (Figure 5). This suggests a delay in the antigen processing under high glucose conditions. Figure 5c shows the phagosome–lysosome by Rab 7, cathepsin D, and *M. tuberculosis* co-localization in low glucose concentrations at 60 min of processing. This was undetected in the high glucose conditions (data not shown).

### 3.6. High Glucose Concentrations Impair M. tuberculosis Antigenic Presentation

**Figure 6:**
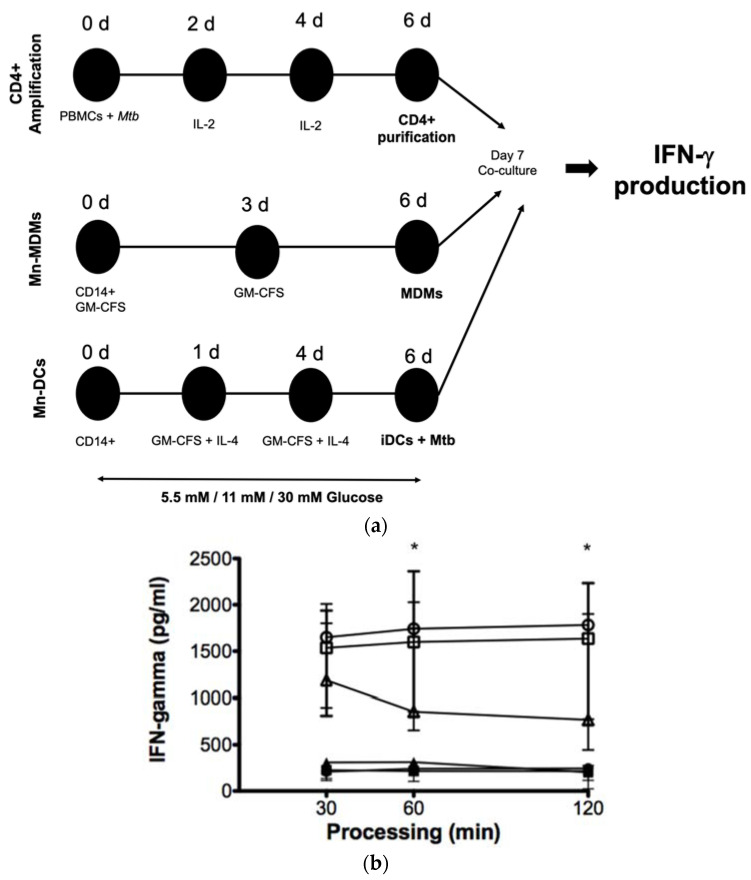
Effect of high glucose concentrations on the processing and presentation of *M. tuberculosis* antigens by MDMs. (**a**) PBMCs isolated from healthy donors were infected with the nonvirulent laboratory strain of *Mycobacterium tuberculosis* *Mtb* H37Ra at a MOI 1:10 at day 0, and IL-2 was added as indicated to amplify specific CD 4+ T cell clones. *Mtb* specific CD4+ T cells were purified by negative selection at day 6. CD14+ cells purified from the same donors at day 0 were differentiated for 6 days to MDMs and DCs as described. MDMs and DCs were cultured in 5.5 mM, 11 mM, 30 mM of glucose and infected with *Mtb* H37Ra at a MOI 1:10 for 1 h and co-cultivated with autologous CD4+ specific T cells for 4 h. Cells and supernatants were collected and IFN-*𝛾* production was analyzed by ELISA. (**b**) Concentration of IFN-γ in the supernatants of co-cultured CD4+ T cells in the presence of 5.5 (open circles), 11 (open squares), and 30 mM (open triangles) of glucose for 6 days infected with and without Mtb infection in 5.5 (filled circles), 11 (filled squares), and 30 mM (filled triangles) of glucose, assayed by ELISA. Data were analyzed using the Friedman test. Data were expressed as mean ± standard deviation. Differences were considered significant when * *p* < 0.01; from five independent experiments.

We then evaluated the effect of 5.5 mM, 11 mM, and 30 mM glucose on the processing and presentation of *M. tuberculosis* antigens in the MDMs. To accomplish that, we used a previously stablished model to study the antigenic presentation in human primary cells in which autologous CD4+ T cells, specific for mycobacterial antigens, were amplified and co-cultured with the MDMs from the same donors. The MDMs were previously exposed to different glucose concentrations and infected with Mtb H37Ra (Figure 6a). As shown in Figure 6b, the IFN-γ production by autologous *M. tuberculosis*-specific T-cells was lower when stimulated with the MDMs cultured in 30 mM glucose when compared with 5.5 mM and 11 mM glucose after 60 min and 120 min of processing, confirming that the delay of the antigenic processing induced by high glucose concentrations results in an altered antigenic presentation.

**Figure 7:**
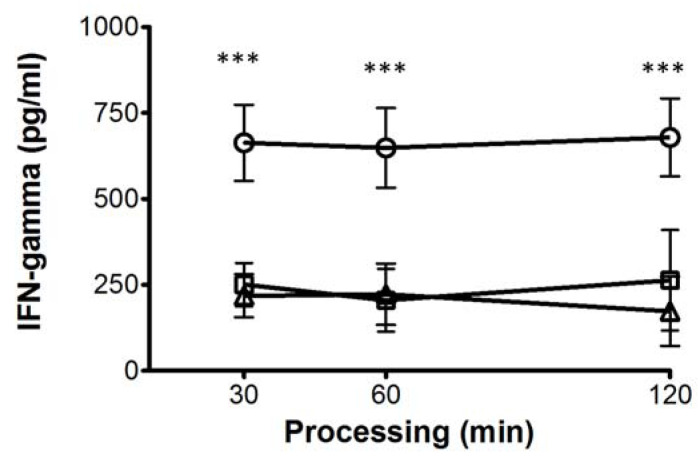
Effect of high glucose concentrations on the processing and presentation of *M. tuberculosis* antigens by DCs. Concentration of IFN-γ in the supernatants of co-cultured CD4+ T cells in presence of 5.5 (open circles), 11 (open squares), and 30 mM (open triangles) of glucose for 6 days infected with *Mtb*, assayed by ELISA. Data were analyzed using Friedman test. Data were expressed as mean ± standard deviation. Differences were considered significant when *** *p* < 0.0001; from five independent experiments.

Moreover, we evaluated the effect of high glucose concentrations in APCs specialized as dendritic cells (DCs). The effect was more pronounced when CD4+ T cells were stimulated with DCs. The decrease in IFN-γ production was clearly observed at 11 mM and 30 mM glucose starting at 30 min of processing, and the effect was maintained up to 120 min of processing (Figure 7), thus suggesting that DCs are more sensitive to high glucose concentrations than the MDMs.

## 4. Discussion

**Figure 8:**
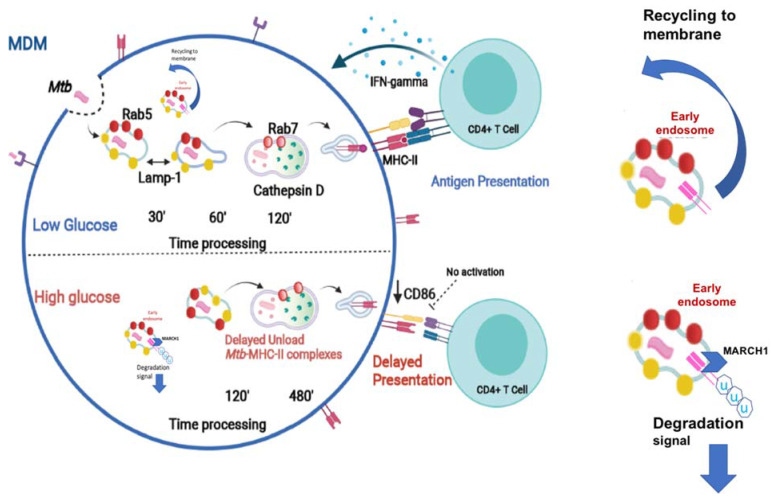
High glucose concentrations modify the traffic of Rab 5, Rab 7, Lamp-1, and cathepsin D, inducing a delay in the *Mtb*–MHC-II complexes formation. An intracellular decrease of the co-stimulatory molecules CD86 and MHC class II molecules is observed, affecting the antigenic presentation. The role of hyperglycemia on MARCH 1-mediated MHC-II protein degradation deserves further exploration. (www.biorender.com).

T2D and pulmonary TB frequently concur in a large number of patients worldwide [33,34]. The immune responses driving the susceptibility of T2D patients to TB are not well understood. This susceptibility is not only attributed to factors such as hyperglycemia and insulin resistance, but also to the response of immune cells to *M. tuberculosis* antigens in the presence of high glucose concentrations [19,35]. In this work, we examined the effect of high glucose concentrations on the mycobacterial antigen processing and presentation. An effective CD4+ T cell activation relies on the adequate antigen presentation by the peptide–MHC II complexes, along with expression of co-stimulatory molecules by the APCs. The vast majority of studies have reported that excess glucose negatively impacts the activity of the MHC class I molecules [36,37]. However, the effect of high glucose concentrations on the functionality of the MHC II molecules has not been explored in depth. Here, we describe that high glucose concentrations diminished both the intracellular HLA-DR and CD86, and that this reduction resulted in the impairment of the antigen presentation by the MDMs, and thus of CD4+ T cell activation. The activity of E3 ubiquitin ligase membrane-associated RING-CH1 (MARCH1) is required to downregulate the cellular levels of both the MHC II and the CD86 molecules [38,39]. Interestingly, MARCH1 is also implicated in insulin receptor degradation and glucose tolerance. The analysis of the relationship between the high glucose concentrations and MARCH activity involved in the phenomenon that we describe would be of prime relevance (Figure 8).

Prior to the presentation, mycobacterial antigens need to be processed; hence, we explored the dynamics of LAMP-1, Rab5, Rab7, and cathepsin D, each of which are key markers of antigen processing.

LAMP-1 is a protein that is associated with the formation of the phagolysosome that mediates *M. tuberculosis* internalization [40]. Our data demonstrate that LAMP-1 expression is unaltered under high glucose concentrations, suggesting that, in our in vitro model, *M. tuberculosis* internalization is unaffected. Rab5 is a GTPase protein in charge of orchestrating the intervesicular trafficking of early phagosomes throughout their fusion with lysosomes. Notably, our results show an abnormal retention of Rab5 until 120 min of high glucose exposure, along with an absence in Rab 7 and cathepsin D acquisition that occurred as late as 480 min of *M. tuberculosis* processing. These findings point to a delay in both traffic and antigen processing in the MDMs.

In normal conditions, lysosomal enzymes such as cathepsin D degrade the invariant chain of MHC II, thus allowing for the formation of peptide–MHC II complexes [41,42].

In line with this evidence, the delay in cathepsin D acquisition under high glucose concentrations concurs with a decrease in HLA-DR, supporting the notion that high glucose concentrations negatively impact the formation of the peptide–MHC II complexes and the antigen presentation. We then performed a functional assay to analyze this effect of excess glucose in antigen processing and presentation on T cell activation by analyzing IFN-γ production. Interestingly, we found that IFN-γ production decreased in lymphocytes co-cultured with the MDMs exposed to high glucose concentrations, confirming that a delayed antigen processing negatively impacts antigen presentation and T cell activation. Moreover, DCs, the most specialized APCs, were more susceptible to high glucose concentrations, inducing less IFN-γ on T cells (supplementary Figure S1). Our findings are in line with the previous information reporting that patients with active TB or T2D show less serum IFN-γ levels than nondiabetic subjects [43].

Several studies in the mice model of streptozotocin-induced diabetes mellitus, and of T2D patients, report an increased CD4+ T cell proliferation but decreased IFN-γ production, exhibiting a dysfunctional phenotype [35,44]. In line with this, we demonstrated that APCs are unable to stimulate appropriate T cells that in turn produce less IFN-γ. Importantly, we found that high glucose concentrations did not affect the intrinsic capacity of CD4+ T cell to proliferate (Figure 2). These results suggest that the MDMs and the DCs are more sensitive to high glucose concentrations than T cells in our in vitro model. Our model allows us to dissect the particular mechanisms that affect each component of the immune response, whereas the in vivo model has many factors that influence the T cell response in T2D.

The endoplasmic reticulum (ER) is the first station of the secretory pathway, and the site of synthesis for proteins resident in the ER or destined for the Golgi compartments, endosomes, lysosomes, the plasma membrane, or the extracellular milieu [45].

Chronic hyperglycemia induces stress of the ER by oxygen species generation and overwhelms the ER protein-folding capacity [46], thus inducing the unfolded protein response and reducing the protein load in the ER by temporarily dropping the global protein synthesis [47,48]. A recent study suggests that, as a homeostatic mechanism, the ER adjusts its secretory flux to the cargo load, and this could alter the arrival of proteins to their final destination [49]. A global stress induced by high glucose concentrations cannot be excluded as an explanation for the impaired phagosome maturation; in fact, other stressors, inducing the unfolded protein response, generate a decline of the MHC class I peptide presentation [50].

In conclusion, our findings provide solid experimental evidence supporting the idea that high glucose concentrations directly affect the phagolysosomal function, resulting in delayed antigen presentation, and thus incomplete CD4+ T cell activation. These findings could explain a mechanism in the susceptibility of patients with diabetes to infection by intracellular pathogens such as *M. tuberculosis.*

## Data Availability

The data presented of this study are available and insert in this article.

## Supplementary Materials

The following are available online at https://www.mdpi.com/article/10.3390/biom11121763/s1, Supplementary Figure S1. Effect of high glucose concentrations on the processing and presentation of *M. tuberculosis* antigens by dendritic cells.
