# Supplementary figures and images for: High Glucose Concentrations Impair the Processing and Presentation of Mycobacterium tuberculosis Antigens In Vitro
Source: Biomolecules. 2021 Nov 25;11(12):1763. doi: 10.3390/biom11121763 (PMC8698639; doi:10.3390/biom11121763)

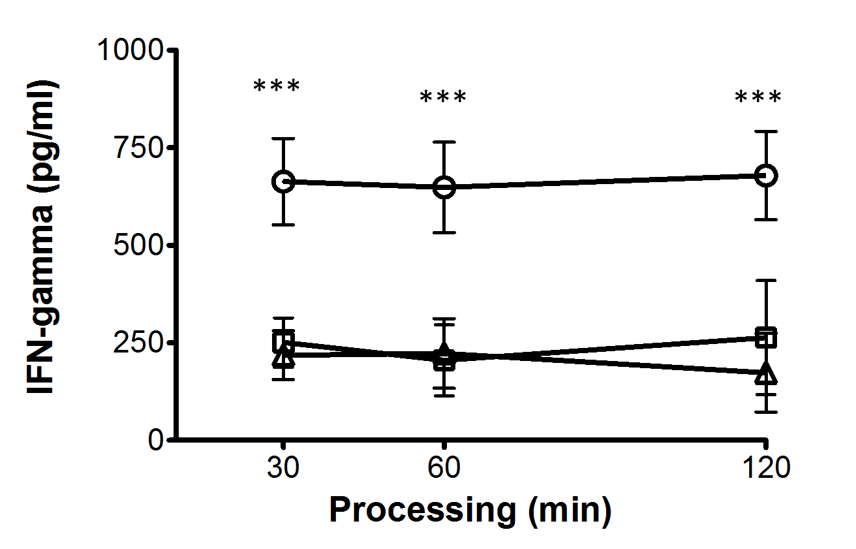

Supplement: Supplementary file 1 [file biomolecules-11-01763-s001.zip › Supplementary Figure S1.tif]
